# Histopathology of Rhizoctonia root and crown rot of sugar beet reveals differential responses in susceptible and resistant cultivars to invasion by *Rhizoctonia solani* AG 2-2

**DOI:** 10.1093/aob/mcag201

**Published:** 2026-07-08

**Authors:** Douglas H Minier, Linda E Hanson

**Affiliations:** Plant, Soil and Microbial Sciences, Michigan State University, East Lansing, MI, USA; Plant, Soil and Microbial Sciences, Michigan State University, East Lansing, MI, USA; United States Department of Agriculture, Sugarbeet and Bean Research Unit, East Lansing, MI, USA

**Keywords:** *Rhizoctonia solani*, *Beta vulgaris*, infection cushion, callose, root anatomy, cell-wall-degrading enzymes

## Abstract

**Background and Aims:**

*Rhizoctonia solani* AG 2-2 causes Rhizoctonia root and crown rot (RRCR) of sugar beet and is a major constraint on production worldwide. Sugar beet roots possess distinctive anatomical features, including a corky periderm, a root groove region and concentric vascular rings, which may influence infection outcomes. Adult-plant resistance is known but the mechanisms are unknown. This study provides an updated histopathology of RRCR and compares responses in resistant and susceptible varieties.

**Methods:**

Six-week-old roots of susceptible (C869) and resistant (SR98/2) varieties were inoculated with *R. solani* AG 2-2 and sampled 2–12 d after inoculation. Tissue sections were examined using brightfield, epi-illumination, fluorescence and confocal microscopy. Stains included aniline blue, wheat germ agglutinin–Alexa Fluor 488, and pectin-specific antibodies LM19 and LM20. Hyphal penetration, infection cushion formation, tissue maceration and cell wall modifications were assessed.

**Key Results:**

Unlike reports from other hosts, hyphae did not align with anticlinal walls or produce T-shaped branches but formed infection cushions through spur branching. Direct penetration occurred without cushions and was reduced on the resistant variety. Hyphae spread laterally between the periderm and outer cambium, with deeper penetration occurring where the outer cambium was disrupted. Tissues within the root groove were associated with structural discontinuities and were especially susceptible. Lesions could be delimited by the presence of red autofluorescence and, in the resistant variety, were bordered by callose. The susceptible variety showed loss of LM19/LM20 epitopes, indicating degradation of methylated and demethylated pectin. Resistant roots retained more LM19/LM20 signal, indicating reduced pectin degradation.

**Conclusions:**

Early infection success is shaped by root anatomy, particularly outer cambium continuity and structures of the root groove. Resistant roots mounted a callose-associated response, whereas susceptible roots exhibited limited cell wall reinforcement. These findings refine the histopathology of RRCR and highlight potential targets for resistance breeding.

## INTRODUCTION

Rhizoctonia root and crown rot (RRCR), caused by *Rhizoctonia solani* AG 2-2, is a major disease affecting sugar beet production in growing regions throughout the world. In severely impacted fields, yield losses can exceed 50 % (Harveson, 2008). Moreover, diseased beets in storage piles can compromise storability and processing quality (Strausbaugh and Gillen, 2008). Above-ground symptoms of RRCR include the sudden and permanent collapse of the leaves, often accompanied by blackening of the petiole at the crown (Windels *et al*., 2009). Root rot symptoms typically begin as dark, circular to oval lesions that often develop in a ladder-like pattern. As the disease progresses, these lesions coalesce and can eventually envelop the entire root (Harveson, 2008). Symptomatic tissue is generally limited to the outer layers of the root during the early stages of disease development and does not penetrate internal tissues until later. It is not uncommon for the entire surface of the beet to become necrotic before disease spreads to the interior of the root (Windels *et al*., 2009). In advanced stages of RRCR, roots may become unharvestable or cause complications during storage and processing (Campbell *et al*., 2014). A dense mat of brownish mycelium may be observed covering internal tissues that are exposed by cracking and cavity formation (Schneider and Whitney, 1986).

*Rhizoctonia solani* is a species complex whose members are classified into anastomosis groups (AGs) based on the ability of their hyphae to anastomose, or fuse, with one another (Cubeta and Vilgalys, 1997). At least 13 AGs have been described (Parmeter *et al*., 1969; Sneh *et al*., 1991), each representing an independently evolving lineage. Anastomosis group 2-2 (AG 2-2) is the primary group responsible for RRCR in adult sugar beet, although other AGs, including AG 4 and AG 5, have also been reported to affect sugar beet (Windels and Nabben, 1989).

Sugar beet (*Beta vulgaris*) is an herbaceous dicotyledonous root crop cultivated for its capacity to accumulate sucrose in the root tissues (Cooke and Scott, 1993). The mature root consists of three regions, the crown, the neck and the true root, with the bulk of the storage tissue located in the true root (Artschwager, 1926). The root is somewhat flattened on two sides that feature prominent, shallow grooves containing the lateral roots. Unlike most herbaceous roots, sugar beet roots lack an epidermis and are instead covered by a thin corky periderm (Artschwager, 1926).

Sugar beet anatomy is unusual among non-woody herbaceous plants (Artschwager, 1926; Elliot and Weston, 1993). In transverse section, the mature root displays concentric growth rings formed by alternating bands of vascular tissue and storage parenchyma (Fig. 1). These rings develop from successive secondary cambium that originate from the pericycle and are initiated in rapid succession, with all rings typically formed within 4–6 weeks. (Artschwager, 1926; Trebbi and McGrath, 2009; Galewski *et al*., 2022).

**Fig. 1.**
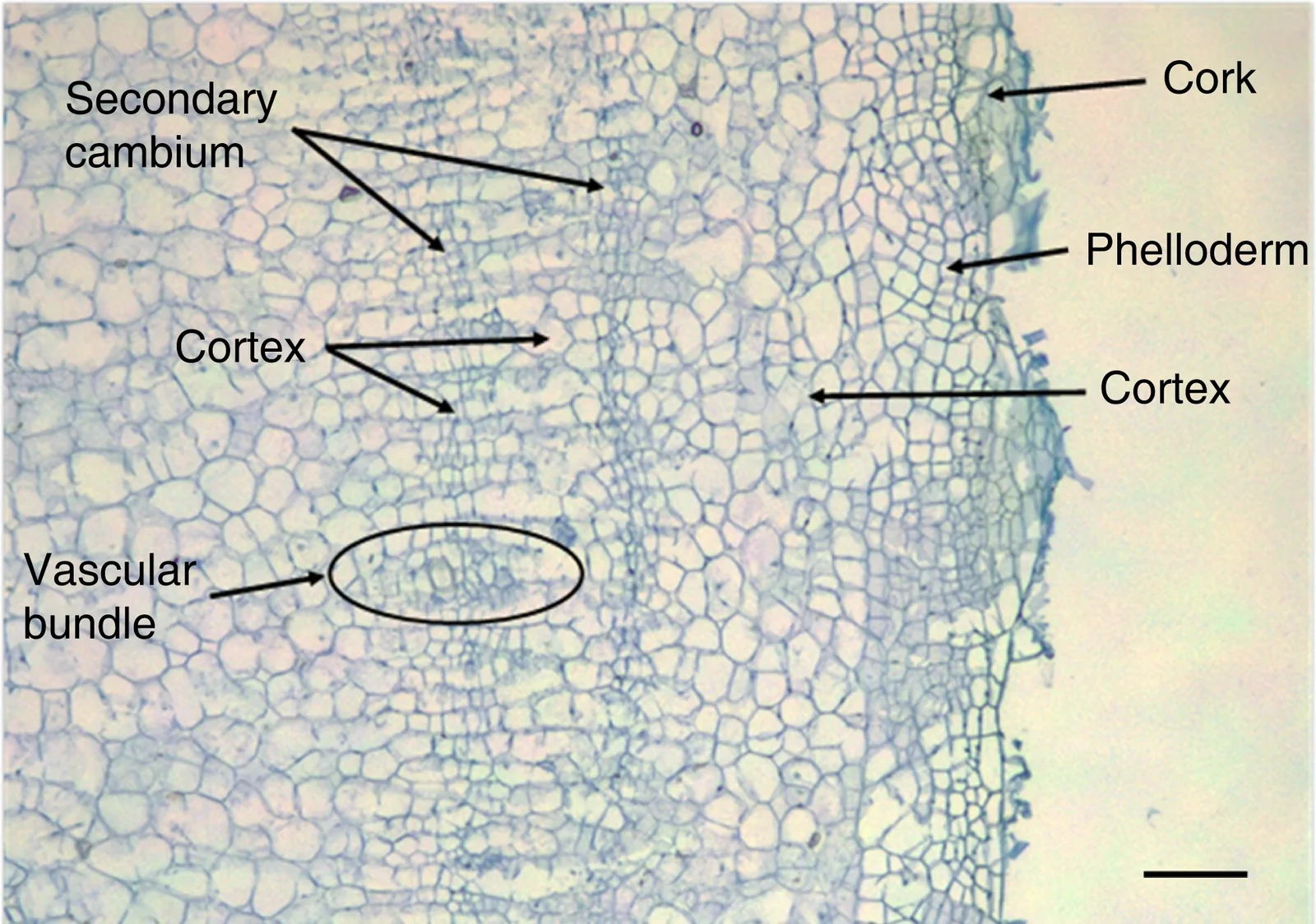
Partial cross-section of 6-week-old sugar beet root of variety C869 showing the outer vascular rings and periderm. The section was stained with toluidine blue O and imaged at 10× with an Olympus BX60 microscope. Scale bar = 100 µm.

The endodermis has been implicated in resistance to Rhizoctonia damping-off during the seedling stage (Nagendran, 2006; Nagendran *et al*., 2009). However, in mature plants, expansion of the root causes the endodermis, the primary cortex and the epidermis to be sloughed off (Artschwager, 1926; Beeckman and De Smet, 2014), and therefore the endodermis does not contribute to adult-plant resistance. In adult roots, the pericycle becomes the outermost persistent tissue and differentiates into phellogen, or cork cambium, which produces the periderm characteristic of mature sugar beet roots (Artschwager, 1926).

Substantial resistance to *Rhizoctonia-*induced diseases typically does not develop until approximately 4–5 weeks after emergence, marking a developmental transition from seedling to adult physiology (Nagendran *et al*., 2009; Liu *et al*., 2019). This transition also coincides with increased sucrose accumulation and completion of vascular ring formation (Trebbi and McGrath, 2009). With the endodermis absent, the outermost secondary cambium becomes the first internal barrier encountered by invading hyphae. Ruppel (1973) observed that *R. solani* hyphae failed to penetrate beyond this outer cambium in resistant varieties. However, no wound periderm formation or suberization was observed. Nonetheless, hyphae spread more rapidly tangentially than radially, pointing to a potential role for the secondary cambium in resistance.

Descriptions of the infection process for *R. solani* come primarily from studies of leaves and other above-ground tissues. In these tissues, infection typically begins as hyphae traverse the host surface, where surface sensing can lead to directed growth along the anticlinal walls and the formation of distinctive T-shaped branches (Armentrout and Downer, 1987; Keijer, 1996; Gonzalez *et al*., 2011). Penetration is initiated by appressoria on several types of foliar tissue (Naito and Sugimoto, 1978; Marshall and Rush, 1980; Zheng and Wang, 2011) or by infection cushions (Keijer, 1996; Zheng and Wang, 2011; Bashyal *et al*., 2018), and their development may be influenced by physicochemical cues, such as waxes, cutin, hydrophobicity and surface hardness (Badri and Vivanco, 2009; Kou and Naqvi, 2016). Penetration may also occur directly through stomata or via infection pegs (Manian and Manibhushanrao, 1982; Zheng and Wang, 2011; Bashyal *et al*., 2018).

Much less is known about the infection process on roots and other underground structures. The surface properties of sugar beet roots differ from above-ground tissues due to the absence of lenticels and stomata and the presence of a corky periderm. Although potato tubers share a similar periderm, potato tubers respond to infection through wound-induced suberization (Lulai, 2007), whereas Ruppel (1973) reported no evidence of suberization following *R. solani* invasion. In cotton, enzymatic degradation of pectic substances beneath infection cushions have been observed prior to penetration (Weinhold and Motta, 1973). In other systems, however, penetration appears to rely primarily on mechanical force rather than enzymatic activity (Armentrout and Downer, 1987; Hofman and Jongebloed, 1988; Yang *et al*., 1992).

Together, these observations underscore the variability of *R. solani* infection strategies across hosts and tissues and support the position that *R. solani* should not be treated as a homogeneous group. That is, processes described for above-ground tissues or other hosts cannot be assumed to apply to sugar beet roots. Despite this need for direct evidence, descriptions of infection processes on sugar beet roots, and roots more generally, are limited.

A histopathological examination of RRCR has not been conducted since Ruppel (1973), despite substantial advances in microscopy and staining techniques. In addition, several RRCR-resistant sugar beet varieties have been developed since 1973, including SR98/2 (PI 655951), the resistant line examined in the present study (McGrath *et al*., 2015). SR98/2, derived from the EL51 breeding line (PI 598074), exhibits resistance to both Rhizoctonia damping-off and RCRR (McGrath *et al*., 2015).

In the current study, we provide an updated histopathological analysis of infection processes associated with RRCR of sugar beet using confocal and advanced digital microscopy. We characterize interactions between *R. solani* AG 2-2 and sugar beet tissues as invasion progresses and identify potential mechanisms involved in RRCR resistance. In addition, our findings help clarify how key physiological features of the root contribute to symptom development observed during infection. This work refines our understanding of pathogen behaviour within host tissues and offers new insight into the development of disease symptoms in resistant and susceptible genotypes.

## MATERIALS AND METHODS

### Sample preparation

Seeds of the sugar beet varieties ‘C869’ (RRCR-susceptible; Lewellen, 2004) and ‘SR98/2’ (RRCR-resistant; McGrath *et al*., 2015) were surface sanitized in 0.6 % sodium hypochlorite with 0.1 % Tween 20 for 15 min, followed by two brief rinses in sterile water. To reduce germination variability, sanitized seeds were soaked overnight in 0.3 % hydrogen peroxide (McGrath *et al*., 2000). Seeds were treated with metalaxyl (Allegiance-FL; Bayer Crop Science, Research Triangle Park, NC, USA) to manage Pythium damping-off and planted in a soilless potting mix (Suremix Pearlite; Michigan Grower Products, Galesburg, MI, USA) in 15-cm-diameter plastic pots. Plants were grown in a controlled environment chamber (Conviron PGW 36, Controlled Environments Inc., Pembina, ND, USA) maintained at 23 °C with a 14-n photoperiod. Osmocote plus fertilizer (ICL Americas, St. Louis, MO, USA) was added to each pot when the plants reached the 4–6-leaf growth stage and plants were watered as needed when the upper 1 cm of the potting mix was dry.

Plants were grown to the 8–10-leaf growth stage and inoculated with isolate ‘R1’ (AG 2-2IIIB) using a single infested barley grain (Gaskill, 1968; Nagendran *et al*., 2009). Two roots from each variety were harvested at 2, 3, 4, 5, 7, 9 and 12 d post-inoculation and gently rinsed with tap water. One uninoculated root for each variety was also harvested at each time point, evaluated for symptoms and processed in a similar manner as the symptomatic tissue to serve as a negative control. In total, about 120 samples were selected and processed through fixation and embedding.

Symptomatic tissue was excised into 3- to 5-mm cuboid sections and fixed in 10 % neutral buffered formalin (Sigma Aldrich) with continuous shaking for 4 d (Fisher *et al*., 2008). Samples were rinsed with 70 % ethanol and stored in 70 % ethanol until embedding. Fixed tissues were embedded in paraffin wax at the Investigative HistoPathology Laboratory (Michigan State University, East Lansing, MI, USA). Sections were cut to a thickness of 12–15 µm on an 820 Histocut microtome (Reichert Technologies, Buffalo, NY, USA), mounted onto glass slides and dried at 57 °C overnight in an incubator.

Prepared sections were dewaxed using a xylene/ethanol rehydration series as described by Nagy *et al*. (2007). The protocol included two changes of Neo-Clear (Sigma Aldridge), one change of histological grade xylenes (Sigma Aldridge), two changes each of 100 and 95 % ethanol, one change each of 70 and 50 % ethanol and, finally, distilled water. Each step was performed for 8 min, and the samples were air dried after the xylenes treatment to minimize contamination of subsequent ethanol washes. Dewaxed slides were dried at 35 °C overnight in an incubator. Reagents used for tissue staining described in the following sections are indicated in Table 1.

**Table 1.** Reagents used in the current study to label cell wall components of sugar beet or *Rhizoctonia solani*.

Reagent	Target	Excitation wavelength (nm)	Emission filter	Notes
Toluidine blue O	General cell wall stain	–	–	Polychromatic stain (O’Brien *et al*., 1964)
Ruthenium Red	Pectin	–	–	(Luft, 1971)
Aniline blue WS	Callose	405	500	Binds to 1-3,β-glucans (Smith and McCully, 1978)
Berberine hemisulfate	Suberin, lignin	405	520	(Brundrett *et al*., 1988)
Wheat-germ agglutinin – Alexa fluor 488	Chitin	488	500	Binds to *N*-acetyl-D-glucosamine (Allen *et al*., 1973)
Safranin O	Lignin, suberin, cutin	–	–	(Kitin *et al*., 2020)
Calcofluor white	Cellulose, chitin	380	475	Binds to 1-3,β- and 1-4,β-polysaccharides in cellulose and chitin (Monheit *et al*., 1986)
Propidium iodide	Cell wall pectin	535	615	Preference for unesterified pectin (Rounds *et al*., 2011)
LM19 rat IgM monoclonal antibody	Unesterified pectin	–	–	Binds to α-1,4 galacturonic acid residues of unesterified pectin (Verhertbruggen *et al*., 2009)
LM20 rat IgM monoclonal antibody	Methyl esterified pectin	–	–	Binds to α-1,4 galacturonic acid residues of esterified pectin (Verhertbruggen *et al*., 2009)
Alexa Fluor 488 goat anti-rat IgG (H + L)	LM19, LM20 primary antibody	488	500	Secondary antibody binds to rat IgM primary antibody (Thermo Fisher)

### Epi-illumination microscopy

Epi-illumination was performed using a VHX-6000 digital microscope (Keyence Corporation of America, Itasca, IL, USA). To enhance visualization of the tissues within the root groove, fresh root cross-sections were cut freehand and stained with 0.05 % (w/v) Ruthenium red (Sigma Aldrich) for 2 min. Samples were rinsed three times in distilled water and imaged within 24 h.

To visualize surface hyphae and the formation of infection cushions, fresh, non-inoculated root sections were harvested, rinsed under running tap water and surface disinfested in a solution of 0.6 % sodium hypochlorite with 0.1 % Tween 20 for 2 min, followed by one rinse in sterile distilled water. Sections were cut into approximately 5-mm square pieces and placed on 2 % (w/v) water agar plates with the periderm oriented up. A 4-mm plug from an actively growing culture of isolate ‘R1’ was placed approximately 1 cm from each root section and allowed to grow onto the tissue. After hyphae visibly made contact, pieces were removed from the agar and stained with lactophenol blue solution (Sigma Aldrich). Sections were visualized on the same day using epi-illumination.

### Brightfield microscopy

General tissue staining was performed using 0.05 % (w/v) toluidine blue O (Sigma Aldrich) in phosphate buffered saline (PBS; Cold Spring Harbor Protocols, 2007). Sections were stained for 2–3 min, rinsed twice with distilled water (O’Brien *et al*., 1964), and dried at 40 °C for 1 to 2 h in an incubator. Cover slips were mounted using Eukitt quick-hardening mounting medium (Sigma Aldrich). Images were acquired on an Olympus BX60 microscope (Evident Corporation, Tokyo, Japan) equipped with an Insight 12-MP CMOS camera (SPOT Imaging, Sterling Heights, MI, USA) and SPOT Advanced v.5.6 image-processing software (SPOT Imaging). The microscope was outfitted with apochromatic objectives Plan 10×/0.25 phi, Plan 20×/0.40 phi and UPlanFl 40×/0.75 ph2. Scale bars were generated using a custom macro that converted pixel measurements to micrometres.

### Confocal and epifluorescence microscopy

Staining with berberine hemisulfate (Sigma-Aldrich) was performed following the protocol of Brundrett *et al*. (1988), using 0.1 % (w/v) berberine hemisulfate in distilled water for 30 min, followed by two rinses in distilled water. Samples were counter stained with 0.5 % (w/v) aniline blue WS (Sigma-Aldrich) in distilled water, rinsed twice in distilled water and mounted in a drop of 0.1 % (w/v) ferric chloride (Sigma Aldrich) in 50 % glycerin. Coverslips were immediately sealed with clear nail polish. Samples stained with aniline blue alone were prepared similarly, but without berberine hemisulfate or ferric chloride treatments, allowing specific visualization of callose tissue (Smith and McCully, 1978). Images were collected with the Olympus BX60 fluorescence microscope, using a DAPI excitation filter (band pass 352–402 nm).

Autofluorescence was assessed by imaging unstained sections using epifluorescence with a dual FITC/TxRed excitation filter (band pass 460–490 and 532–554 nm). Additionally, unstained sections were examined using an A1Rsi laser scanning confocal microscope (A1Rsi LSCM; Nikon Instruments Inc., Melville, NY, USA) using 408-, 489- and 561-nm diode lasers and the band pass filters 430–480, 500–550 and 570–620 nm, respectively. Image scanning was performed using unidirectional Galvano scanning with a gallium arsenide phosphide (GaAsP) PMT detector. The pinhole aperture was automatically set to 1 Airy Unit. Z-stacks were acquired using ten optical slices at 1-µm steps, and area scans were collected with a 10 % overlap.

For safranin O/calcofluor white staining, samples were immersed in a 0.1 % (w/v) aqueous solution of safranin O (Sigma Aldrich) for 3–5 min and de-stained by sequential rinsing in 25, 50, 70 and 95 % ethanol for 10 min each, until dye no longer leached from the samples. Samples were rehydrated by reversing through the ethanol series (70, 50 and 25 %) for 10 min each, followed by distilled water (Kitin *et al*., 2020). One drop of 10 % potassium hydroxide was applied to each sample, followed by two drops of Calcofluor white stain (0.1 % calcofluor white M2R plus 0.05 % Evans blue; Sigma Aldrich). After a 5-min incubation at room temperature, samples were rinsed twice in distilled water, dried at 40 °C for 1–2 h in an incubator and mounted using Eukitt quick-hardening mounting medium (Sigma Aldrich). Imaging was performed using epifluorescence on an Olympus BX60 fluorescence microscope, using a triple DAPI/FITC/TxRed excitation filter (band pass 352–402, 460–490 and 532–554 nm).

Fungal hyphae within sugar beet root tissue were visualized using fluorescence techniques adapted from Carotenuto and Genre (2020). Samples were cleared by applying a solution of 0.6 % sodium hypochlorite with 0.1 % Tween 20 directly to the slide for 5 min, followed by three rinses in PBS. Cleared tissues were incubated for 1 h in wheat germ agglutinin (WGA) conjugated with Alexa Fluor 488 (10 mg mL^–1^ in PBS; Thermo Fisher, Waltham, MA, USA). After three rinses in PBS, two drops of propidium iodide (100 ng mL^–1^; Sigma-Aldrich) in PBS were applied for 1–2 min to counterstain cell walls. Samples were rinsed three times with PBS and mounted with Fluoro-Gel mounting medium (Electron Microscopy Sciences, Hatfield, PA, USA). Imaging was performed on an A1Rsi LSCM with either a PlanApo 20× VC (NA 0.75) or a PlanApo λ 10× (NA 0.45) objective. Alexa Fluor 488 fluorophore was excited with a 489-nm diode laser and detected using a 500–550-nm bandpass filter. Propidium iodide was excited with a 561-nm diode laser and detected using a 570–620-nm bandpass filter.

Sections exhibiting changes in autofluorescence were examined for pectin esterification state using the rat monoclonal antibodies LM19 and LM20, which recognize de-esterified and methyl-esterified pectin, respectively (Verhertbruggen *et al*., 2009). Sequential sections were hydrated in PBS for 15 min and cleared with a solution of 0.6 % sodium hypochlorite and 0.1 % Tween 20 for 5 min. Samples were rinsed twice in PBS and incubated with 5 % bovine serum albumin (Sigma Aldrich) to block non-specific binding of primary antibodies (Miller and Shakes, 1995). After two rinses in PBS, samples were incubated with a 1:10 dilution of either the LM19 or LM20 antibody for 30 min. Samples were rinsed in PBS three times and incubated with a 1:100 dilution of anti-rat IgG conjugated to Alexa Fluor 488 (Thermo Fisher) for 20 min. Sections were rinsed three times in PBS and mounted using Fluoro-Gel mounting medium. Imaging was performed on the A1Rsi LSCM equipped with an Apo 60× Oil λS (NA = 1.40) objective. Alexa Fluor 488 was excited with a 489-nm diode laser with a 500–550-nm band pass emission filter.

## RESULTS

### Infection cushion formation

The external cork cells of the sugar beet root tissue were irregularly shaped and loosely arranged in rows. Surface hyphae did not follow the anticlinal walls; instead, runner hyphae crossed the root surface at oblique angles and branched at regular intervals (Fig. 2). Although some branch spurs did orient along anticlinal walls, this behaviour was inconsistent and was not the predominant behaviour. T-shaped branching was not observed. Rather, lateral hyphae produced progressively shorter spurs that formed dense hyphal aggregates (Fig. 2). These aggregates were recognizable as infection cushions, and were composed of thick-walled, highly interconnected cells with a strong affinity for lactophenol blue (Fig. 2).

**Fig. 2.**
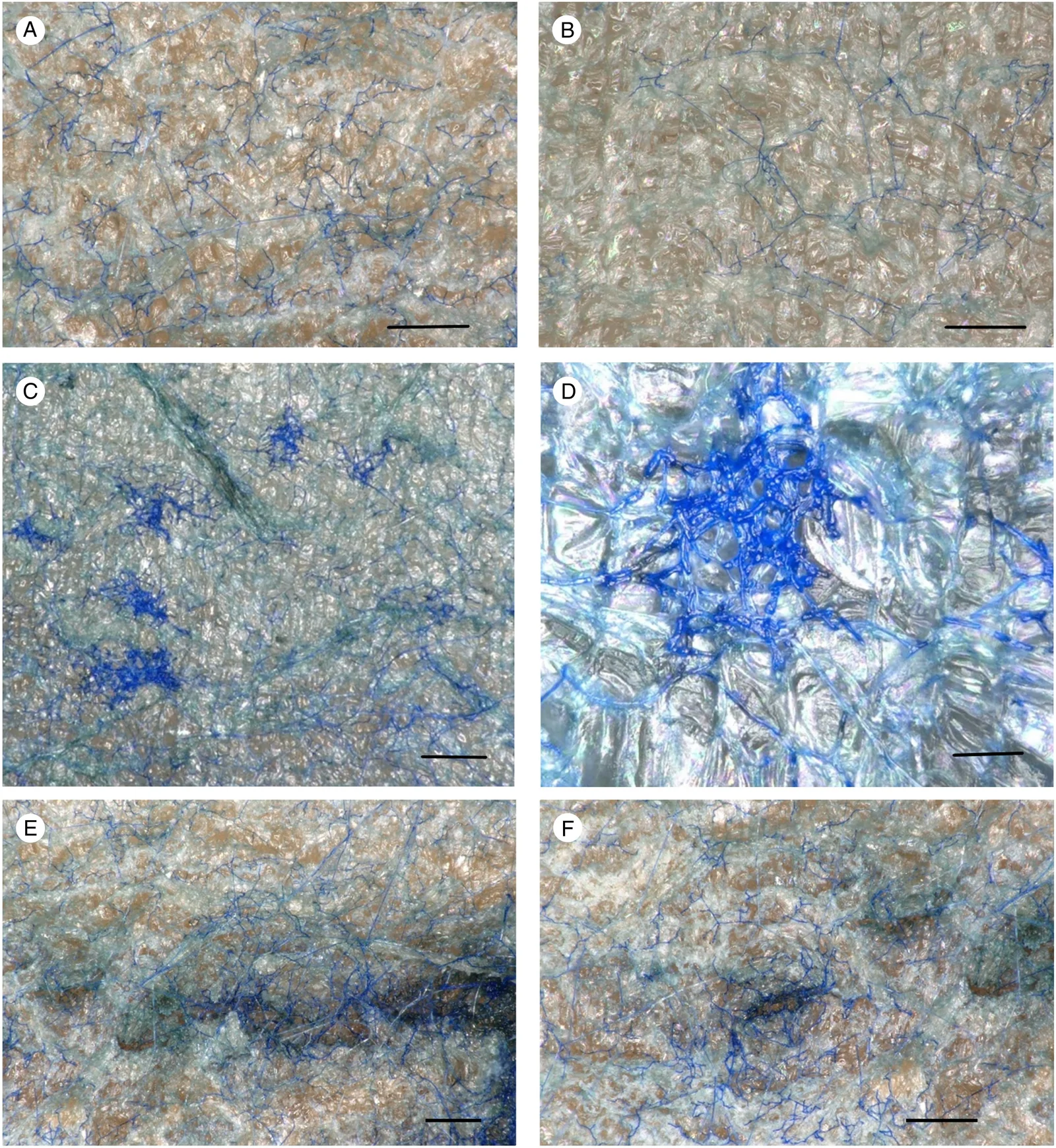
Hyphae of *Rhizoctonia solani* AG 2-2 on the surface of root sections from 6-week-old sugar beet plants. Hyphae were stained with lactophenol blue 3 d after inoculation and imaged with a Keyence VHX6000 digital microscope. Hyphe traversed the surface at oblique angles on (A) the susceptible variety C869 (200×) and (B) the resistant variety SR98/2 (200×). Infection cushions are shown forming on the susceptible variety C869 at (C) 100× and (D) 500×. Hyphae aggregate around natural openings of the susceptible variety C869 and are shown at (E) 150× and (F) 200×. Scale bars for images A, B, C, E and F = 250 µm and for image D = 50 µm.

No distinct surface features were consistently associated with infection cushion formation, although hyphae occasionally accumulated around wounds or natural cracks in the cork layer (Fig. 2). Infection cushions typically formed at sites where two or more hyphae overlapped, and their frequency was related to the density of the surface hyphae and extent of spur branching. Surface hyphae were denser on the susceptible variety than on the resistant variety, and infection cushions were correspondingly more abundant. On the resistant variety, infection cushions were rarely observed (Fig. 2).

Formation of infection cushions did not appear to be necessary for penetration of the outer cork cells. Multiple instances were observed in which hyphae directly penetrated the outer cork layer without an associated infection cushion (Fig. 3). In cross-section, hyphae were present within the outer cork layers and periderm, even in the absence of visible infection structures near the site. The precise mode of penetration into the periderm, whether intercellular or intracellular, could not be determined. However, in cases where penetration occurred beneath an infection cushion, hyphae were observed entering intracellularly and compromising the host cell wall (Figure 4).

**Fig. 3.**
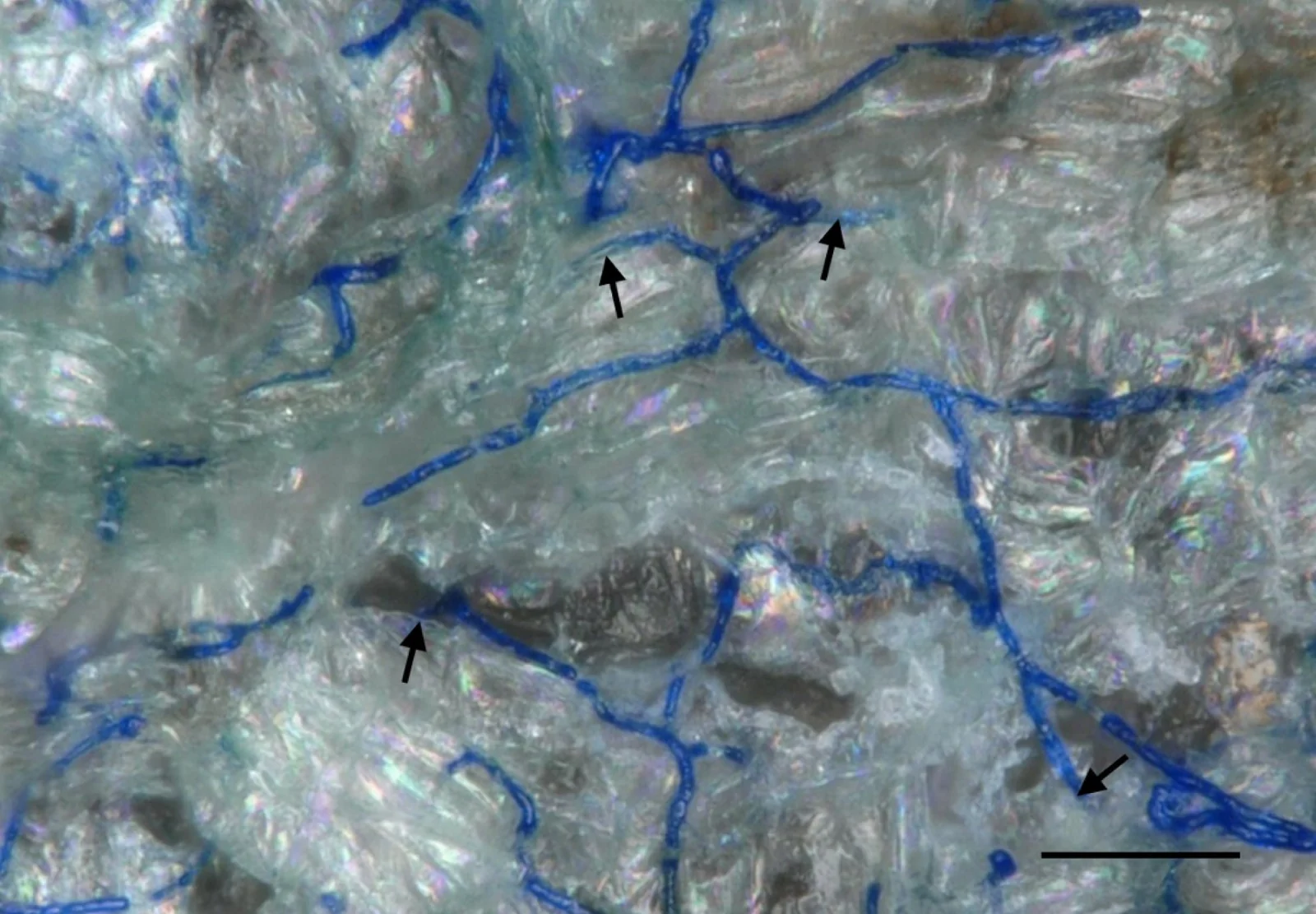
Hyphae of *Rhizoctonia solani* AG 2-2 on the surface of root tissue from 6-week-old sugar beet plants of the susceptible variety C869, 3 d after inoculation. Arrows indicate hyphae directly penetrating the outer cork layer. Hyphae were stained with lactophenol blue and imaged at 1000× with a Keyence VHX6000 digital microscope. Scale bar = 50 µm.

**Fig. 4.**
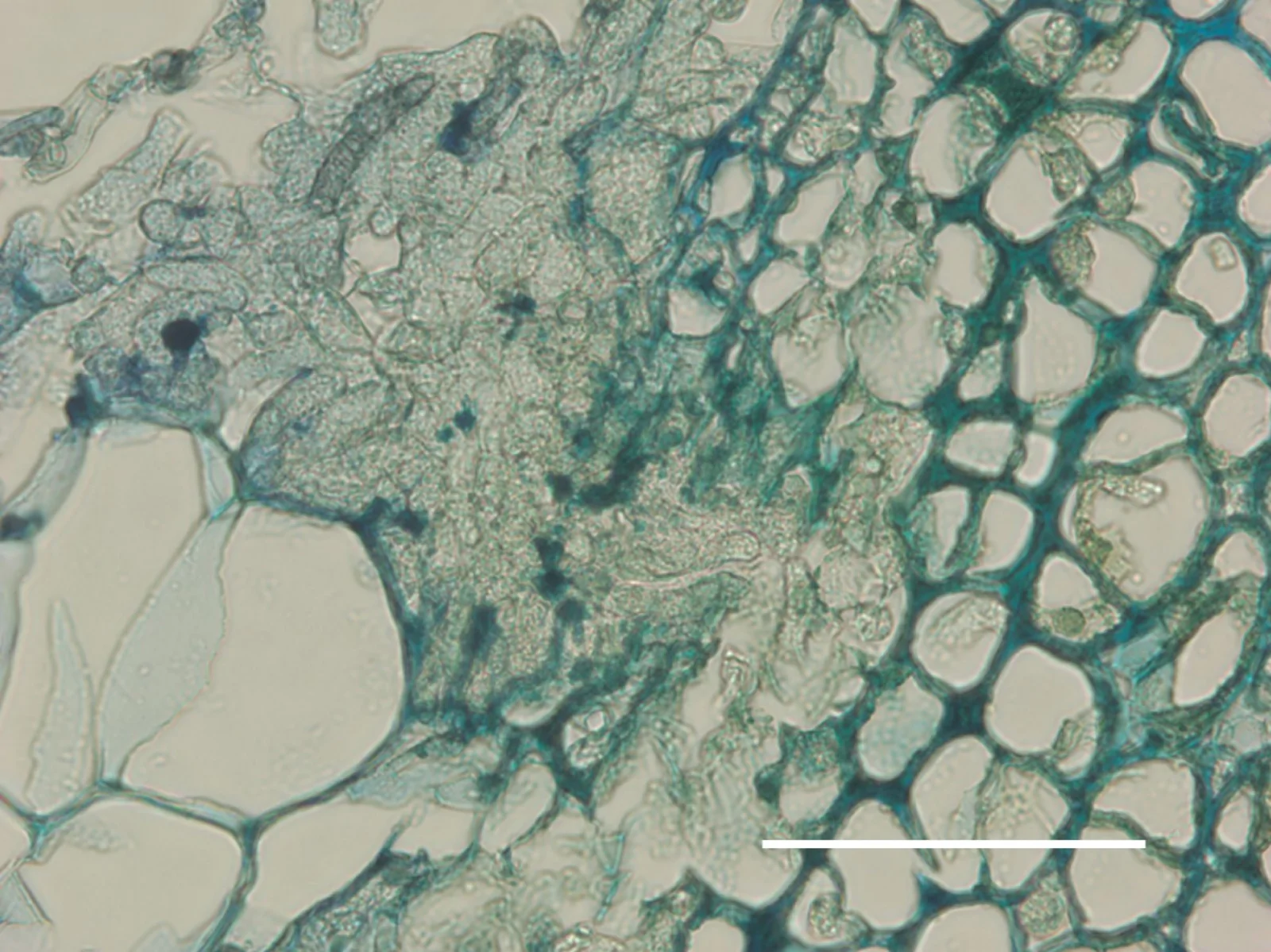
Hyphae of *Rhizoctonia solani* AG 2-2 invading root tissue of a 6-week-old sugar beet plant of the susceptible variety C869 beneath an infection cushion 4 d after inoculation. Imaged at 40× with an Olympus BX60 microscope. Scale bar = 100 µm.

### Progression of fungal invasion

Hyphae that entered through the smooth root surface were detected within the outer cork layer as early as 3 d post-inoculation (Fig. 5). These hyphae did not immediately breach the outermost cambium and instead grew laterally into the cortex region between the periderm and outer cambial layers. Colonization progressed laterally within this region, with deeper penetration into the root occurring where the outer cambium was broken or disrupted (Fig. 5). Parenchyma tissue located between vascular bundles increased in size toward the centre of the root, creating broader zones of cambial disruption where further hyphal advancement was observed.

**Fig. 5.**
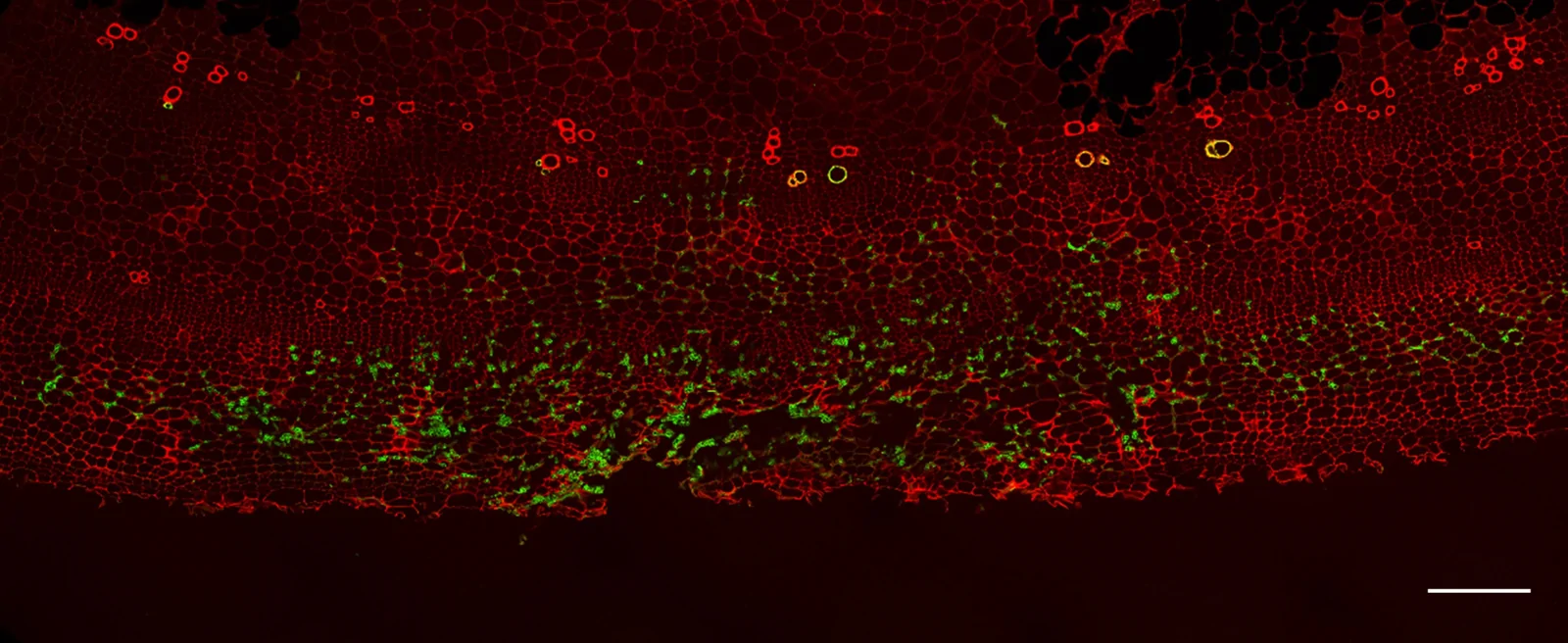
Hyphae of *Rhizoctonia solani* AG 2-2 within root tissue of 6-week-old sugar beet plants of the resistant variety SR98/2, 3 d after inoculation. Fungal hyphae were labelled with wheat germ agglutin conjugated to Alexa Fluor 488 (green) and plant tissue was stained with propidium iodide (red). Imaged at 20× with a Nikon A1Rsi laser scanning confocal microscope. Scale bar = 250 µm.

Maceration was evident in the outer cortex within 3 d post-inoculation. Affected regions appeared visibly darkened and remained distinguishable after sectioning. Within the maceration zone, cell walls were fragmented and disorganized and were associated with localized tissue collapse. Vascular tissue showed little to no degradation compared with the cortex. However, autofluorescence associated with the lesion was not restricted by the vascular tissue and extended into adjacent tissues (Fig. 6).

**Fig. 6.**
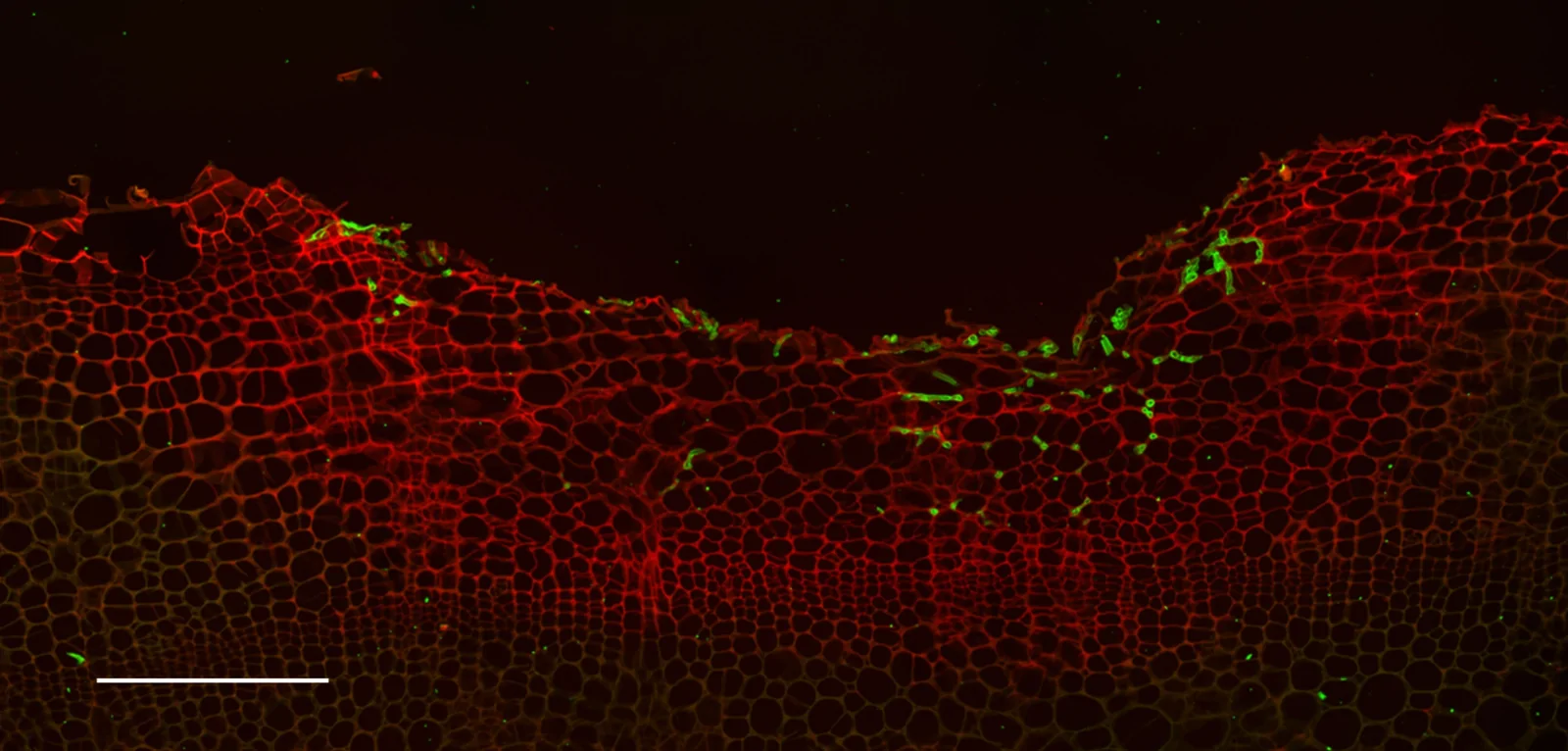
Fluorescence associated with a lesion caused by *Rhizoctonia solani* AG 2-2 in 6-week-old sugar beet roots of the variety C869, 2 d after inoculation. Fungal hyphae were labelled with wheat germ agglutin conjugated with Alexa Fluor 488 (green) and plant tissue was stained with propidium iodide (red). Imaged at 20× with a Nikon A1Rsi laser scanning confocal microscope. Scale bar = 250 µm.

Tissues associated with the root groove and lateral roots form complex folds and grooves (Fig. 7), in which hyphae were observed entering through multiple points (Fig. 8). Hyphae were frequently present within these structural features, but no infection cushions were observed at these penetration sites (Fig. 8). Although cork cells were present on the outer surfaces of the root-groove tissues, the transitions between lateral roots and the main root were irregular, with multiple discontinuities. Hyphae were detected in and around lateral roots and appeared to follow lateral-root tissue into the main root, where subsequent spread was extensive (Fig. 8). In contrast to the continuous outer cambium of the main root, the outer cambium was either absent or structurally disrupted in these regions.

**Fig. 7.**
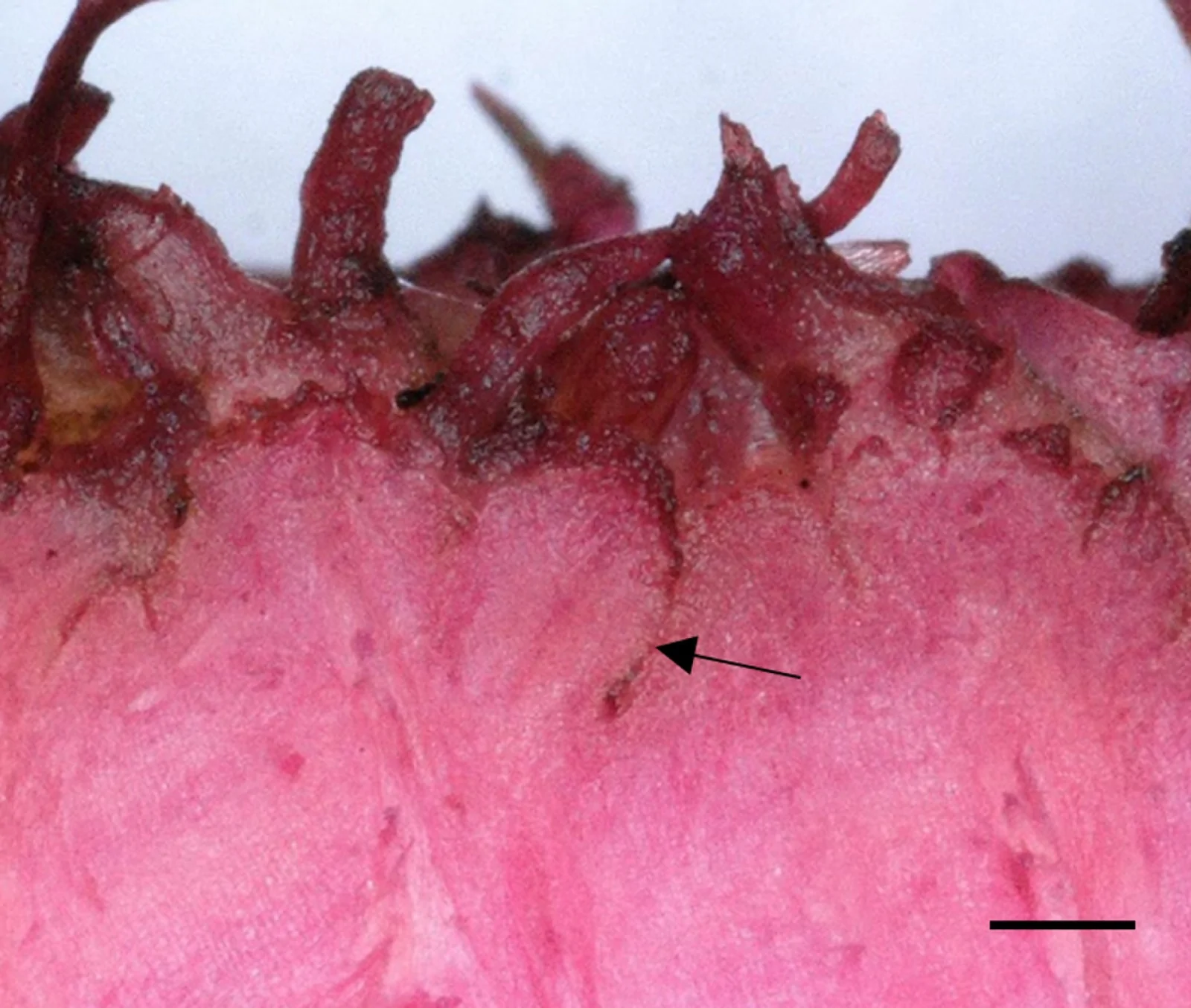
Longitudinal section through the root groove region of 6-week-old sugar beet root of the variety C869 stained with ruthenium red. The arrow indicates a gap between folds of tissue. Imaged at 100× with a Keyence VHX-6000 digital microscope. Scale bar = 500 µm.

**Fig. 8.**
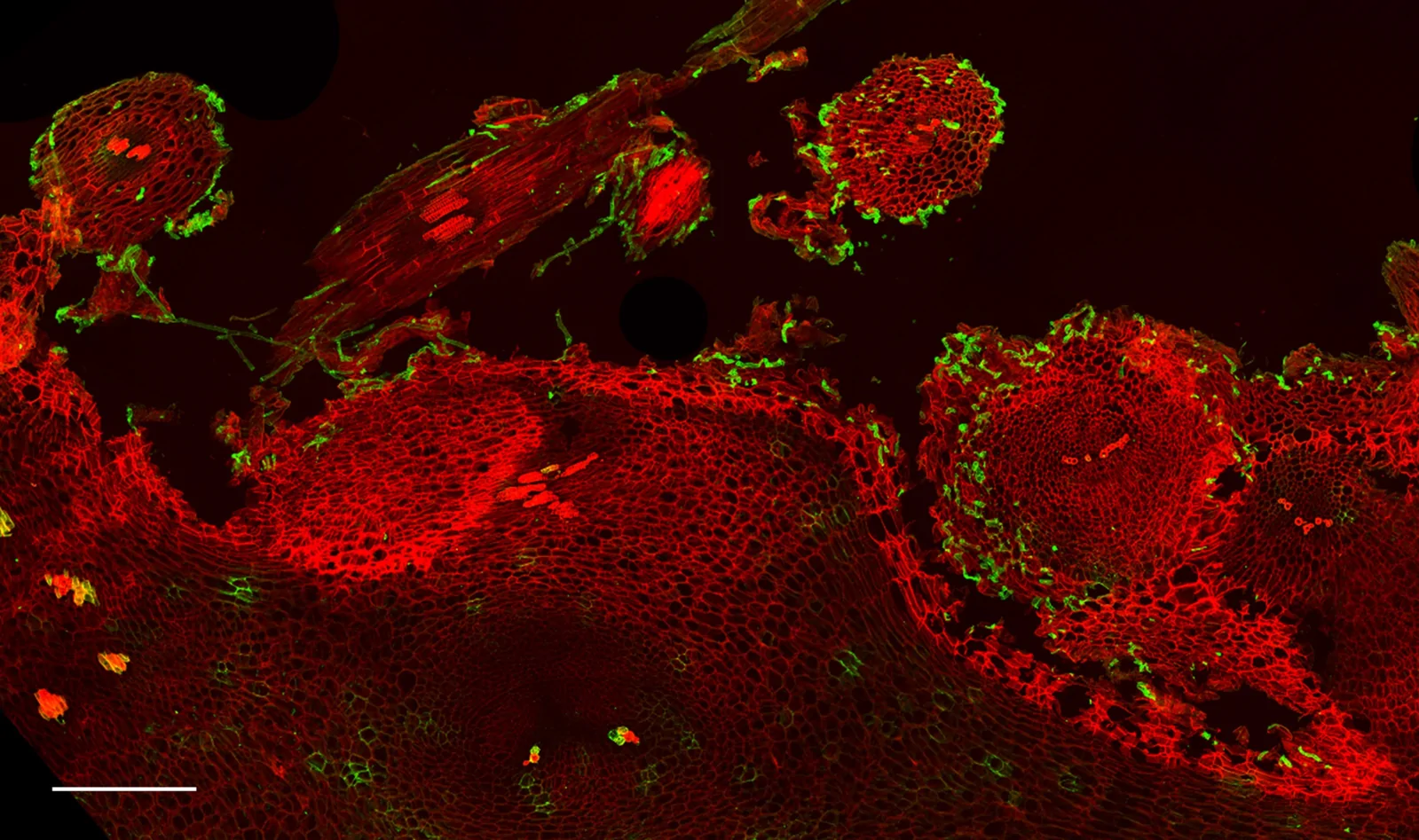
Hyphae of *Rhizoctonia solani* AG 2-2 within the root groove of a 6-week-old sugar beet root of the resistant variety SR98/2, growing among lateral roots, 4 d after inoculation. Fungal hyphae were labelled with wheat germ agglutin conjugated to Alexa Fluor 488 (green) and plant tissue was stained with propidium iodide (red). Imaged at 20× with a Nikon A1Rsi laser scanning confocal microscope. Scale bar = 250 µm.

### Alteration of cell wall components

Cell walls exhibited visually detectable, lesion-associated changes beyond the structural alterations described above. As early as 2 d after inoculation, a distinct zone of discoloration developed around sites of hyphal invasion and extended up to 500 µm beyond the nearest detectable hyphae (Fig. 6). Within this region, unstained tissue displayed strong autofluorescence under FITC/TxRed excitation (bandpass 460–490 and 532–554 nm). Cell walls at the lesion margins appeared thickened and showed elevated fluorescence in the red spectrum (Fig. 9). In areas of the lesion where tissue maceration had progressed, fluorescence intensity was markedly reduced. Both resistant and susceptible varieties exhibited similar autofluorescence patterns; however, the susceptible variety consistently showed broader zones of lesion-associated fluorescence, reflecting more extensive tissue involvement.

**Fig. 9.**
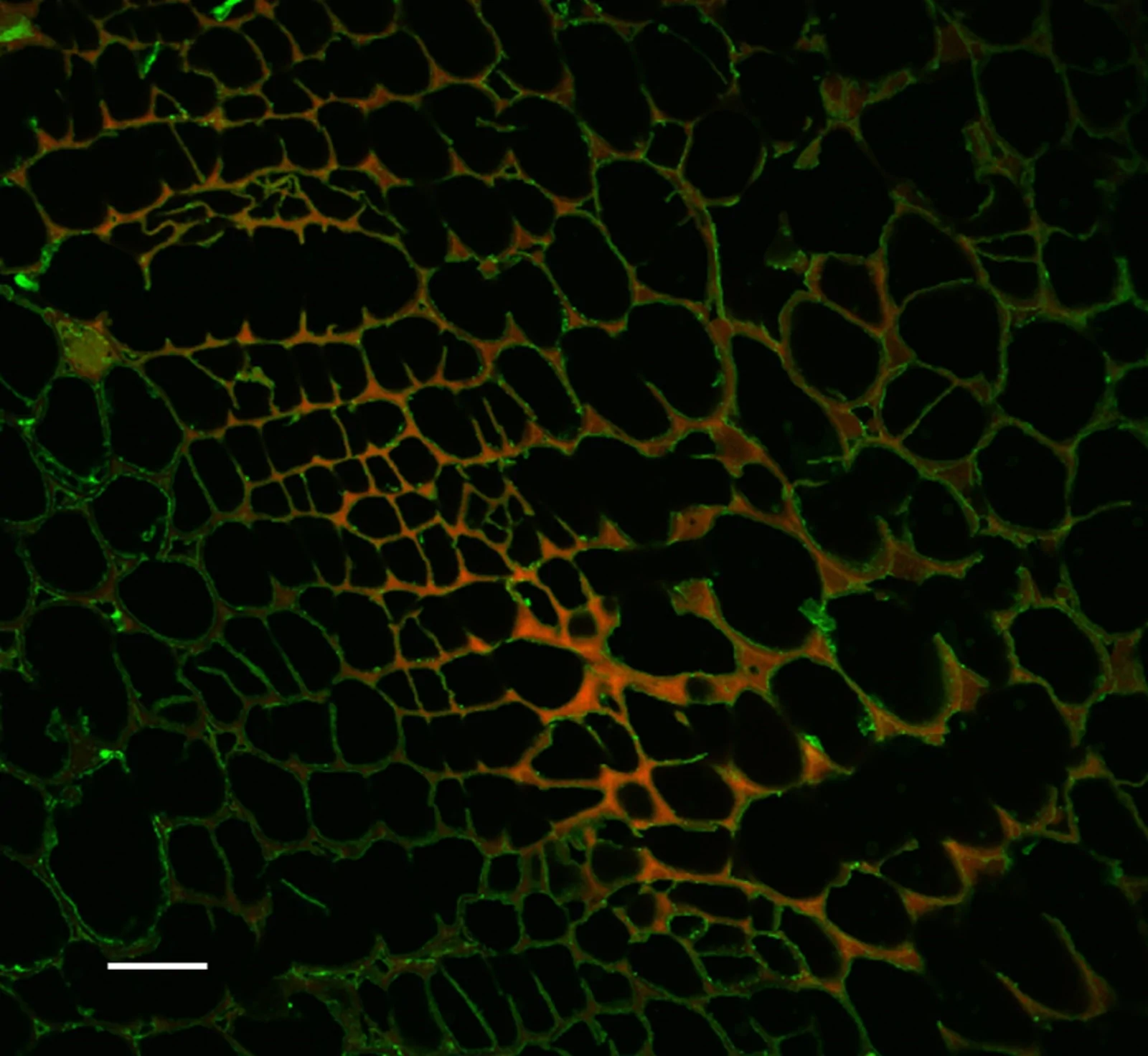
Root tissue from 6-week-old sugar beet root of the variety C869 infected with *Rhizoctonia solani* AG 2-2, 4 d after inoculation, showing thickened cell walls. Tissue was immunolabelled with the monoclonal antibody LM19, and a goat anti-rat IgG secondary antibody conjugated to Alexa Fluor 488 was used as the fluorescent label. Red signal reflects autofluorescence rather than an applied stain. Imaged at 20× with a Nikon A1Rsi laser scanning confocal microscope. Scale bar = 25 µm.

In samples stained with berberine hemisulfate, increased fluorescence was observed at the lesion margins with a stronger signal in the resistant variety than in the susceptible one (Fig. 10). Because berberine hemisulfate binds several cell wall components, additional staining was performed using aniline blue alone to specifically detect callose. The fluorescent pattern observed with aniline blue closely mirrored that of berberine hemisulfate, consistent with callose accumulation at the lesion margins. This response was detectable as early as 4 d post-inoculation, though weak, and became strong and well defined by 9 d post-inoculation in the resistant cultivar. In contrast, the susceptible variety showed a markedly diminished response, with no appreciable signal up to 12 d after inoculation (Fig. 10).

**Fig. 10.**
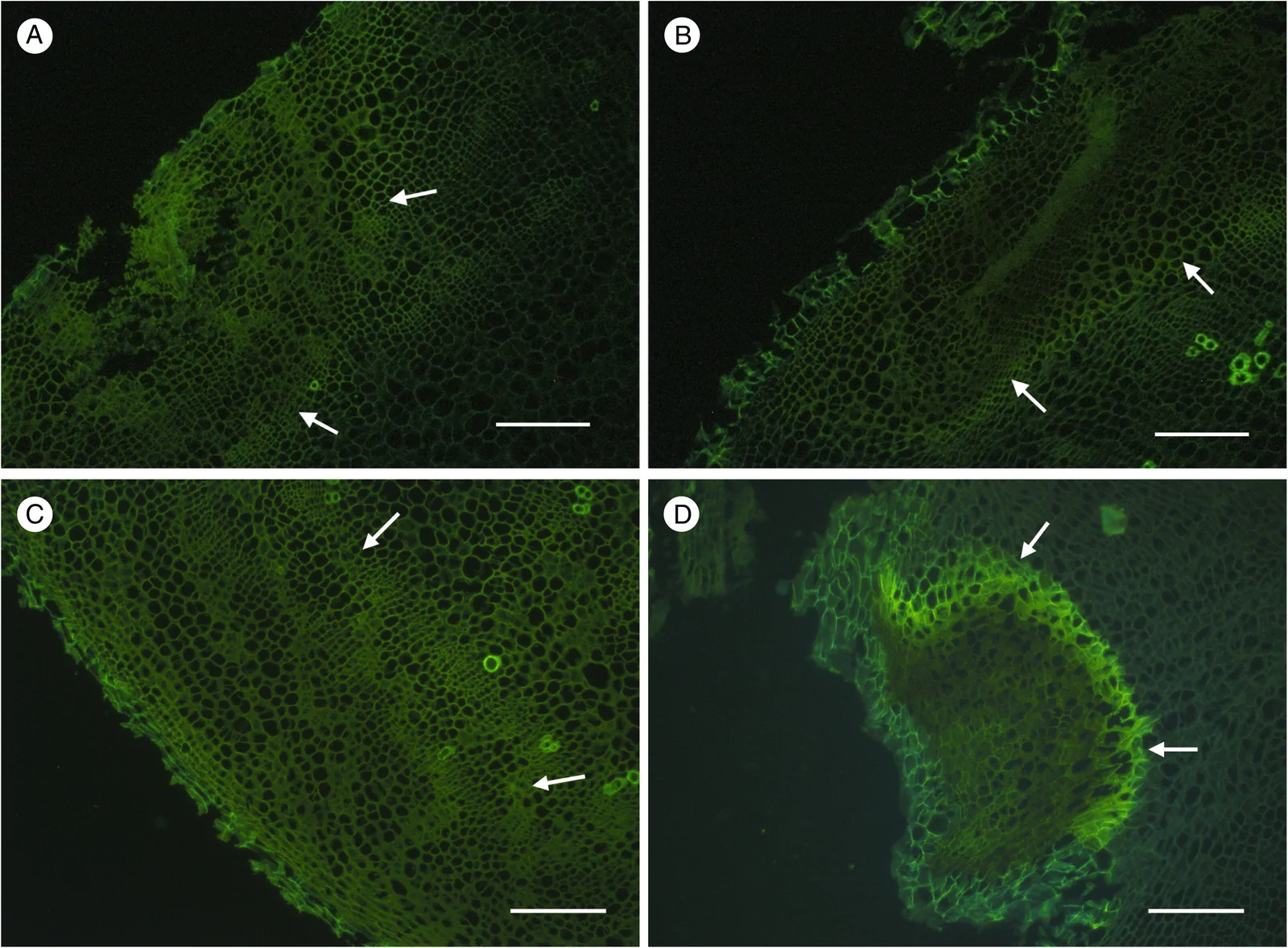
Root tissue from 6-week-old sugar beet plants infected with *Rhizoctonia solani* AG 2-2 and stained with berberine hemisulfate and aniline blue. The susceptible variety C869 is shown in (A) 4 d after inoculation and (C) 12 d after inoculation. The resistant variety SR98/2 is shown in (B) 4 d after inoculation and (D) 12 d after inoculation. Arrows indicate lesion boundaries identified by red autofluorescence (not visible in these panels). Imaged at 10× with an Olympus BX60 fluorescence microscope using a DAPI excitation filter (band pass 352–402 nm). Scale bar = 200 µm.

Safranin O staining was more intense in diseased tissue than in healthy tissue, whereas calcofluor white exhibited reduced fluorescence in infected regions (Fig. 11). This diminished calcofluor white signal indicated a loss or disruption of cellulose within the infected regions. Beyond the lesion margin, safranin O fluorescence diminished but partially masked the weaker calcofluor white signal. In portions of the lesion where tissue maceration had begun, both stains showed markedly reduced binding, with the most severely damaged tissue exhibiting minimal to no fluorescence. Autofluorescence, however, persisted until the tissue was nearly fully degraded. The resistant variety displayed a stronger fluorescence response than the susceptible one, forming a distinct band at the lesion margin similar to the pattern observed with aniline blue staining.

**Fig. 11.**
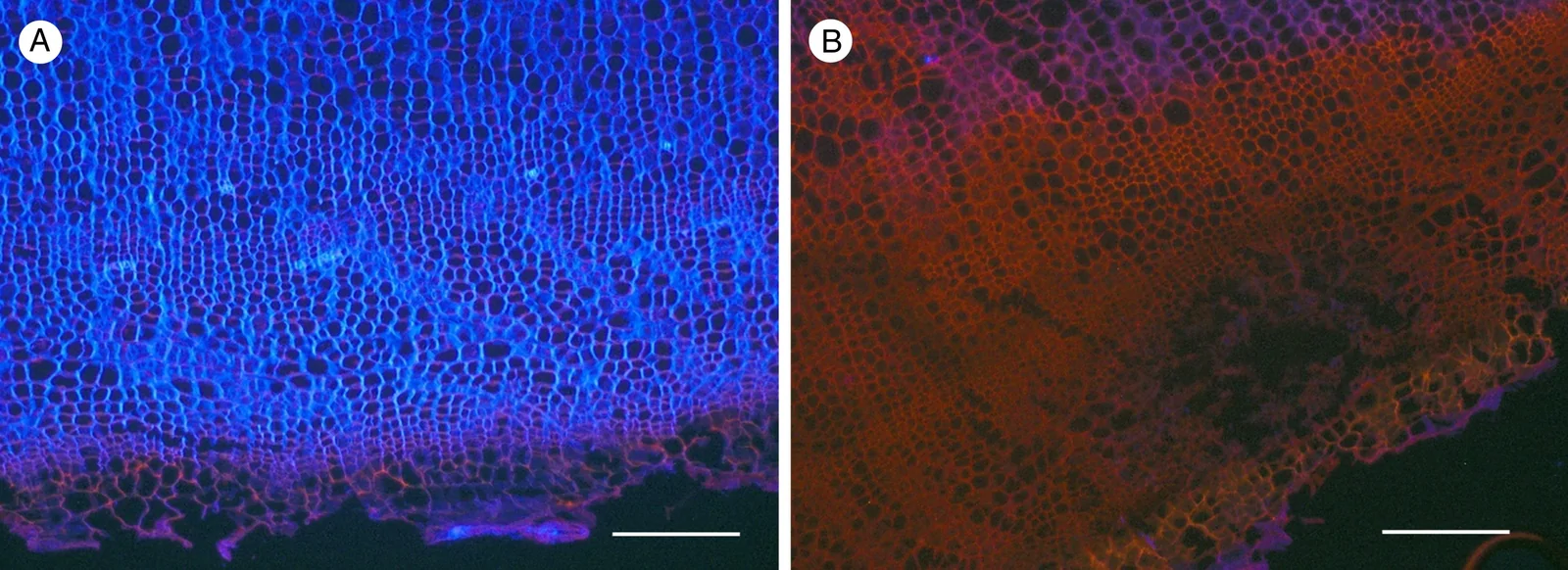
Root tissue from 7-week-old sugar beet plants of the resistant variety SR98/2 stained with calcofluor white and safranin O 7 d after inoculation: (A) uninfected tissue, and (B) tissue inoculated with *Rhizoctonia solani* AG 2-2, 3 d after inoculation. Imaged at 10× with an Olympus BX60 fluorescence microscope using a DAPI/FITC/TxRed excitation filter (band pass 352–402, 460–490, 532–554 nm). Scale bar = 250 µm.

Minimal differences were observed in the distribution of pectic epitopes labelled by LM19 and LM20 antibodies in healthy tissues (Figs 12 and 13). Both pectic epitopes were present in similar quantities, although the LM20-labelled epitope appeared slightly more prominent than the LM19-labelled epitope. In infected tissue, the LM19-labelled epitope was less detectable in the susceptible variety, C869, than the LM20 epitope, which remained visible within the intracellular region (Fig. 12). In the resistant variety, SR98/2, the LM19 epitope showed pronounced aggregation along the inner surface of the cell walls (Fig. 13). The LM20 epitope also exhibited aggregation, but to a lesser extent than LM19. Overall, both epitopes were reduced in infected tissue compared to healthy tissue, but the resistant variety retained more labelled material, particularly for the LM19 epitope (Fig. 13).

**Fig. 12.**
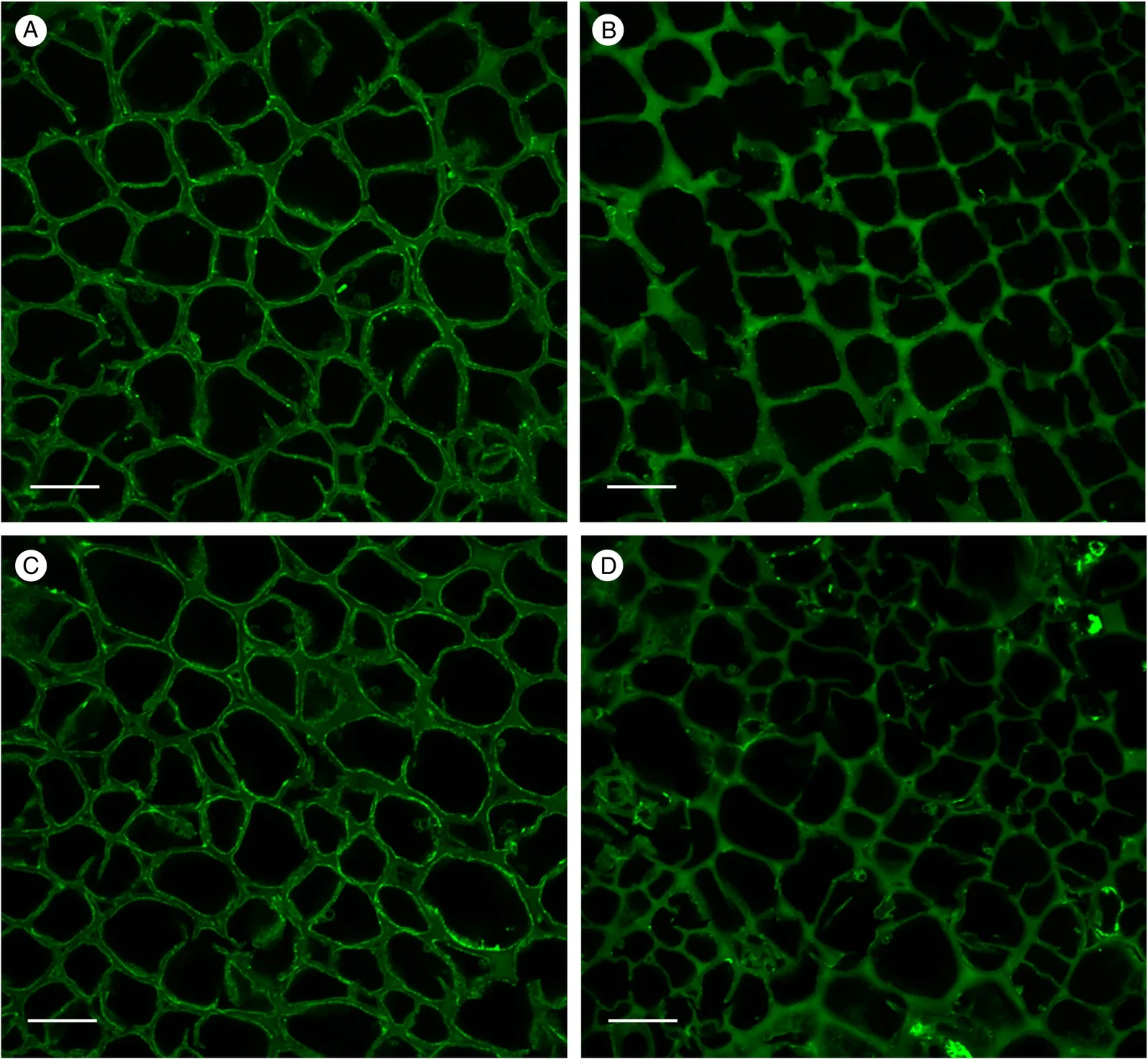
Root tissue from 6-week-old sugar beet plants of the variety C869 immunolabelled with the rat IgM monoclonal antibodies LM19 and LM20, 4 d after inoculation: (A) LM19 healthy, (B) LM19 infected, (C) LM20 healthy and (D) LM20 infected. A goat anti-rat IgG secondary antibody conjugated to Alexa Fluor 488 dye was used as the fluorescent label. Imaged at 40× with a Nikon A1Rsi laser scanning confocal microscope. Scale bar = 25 µm.

**Fig. 13.**
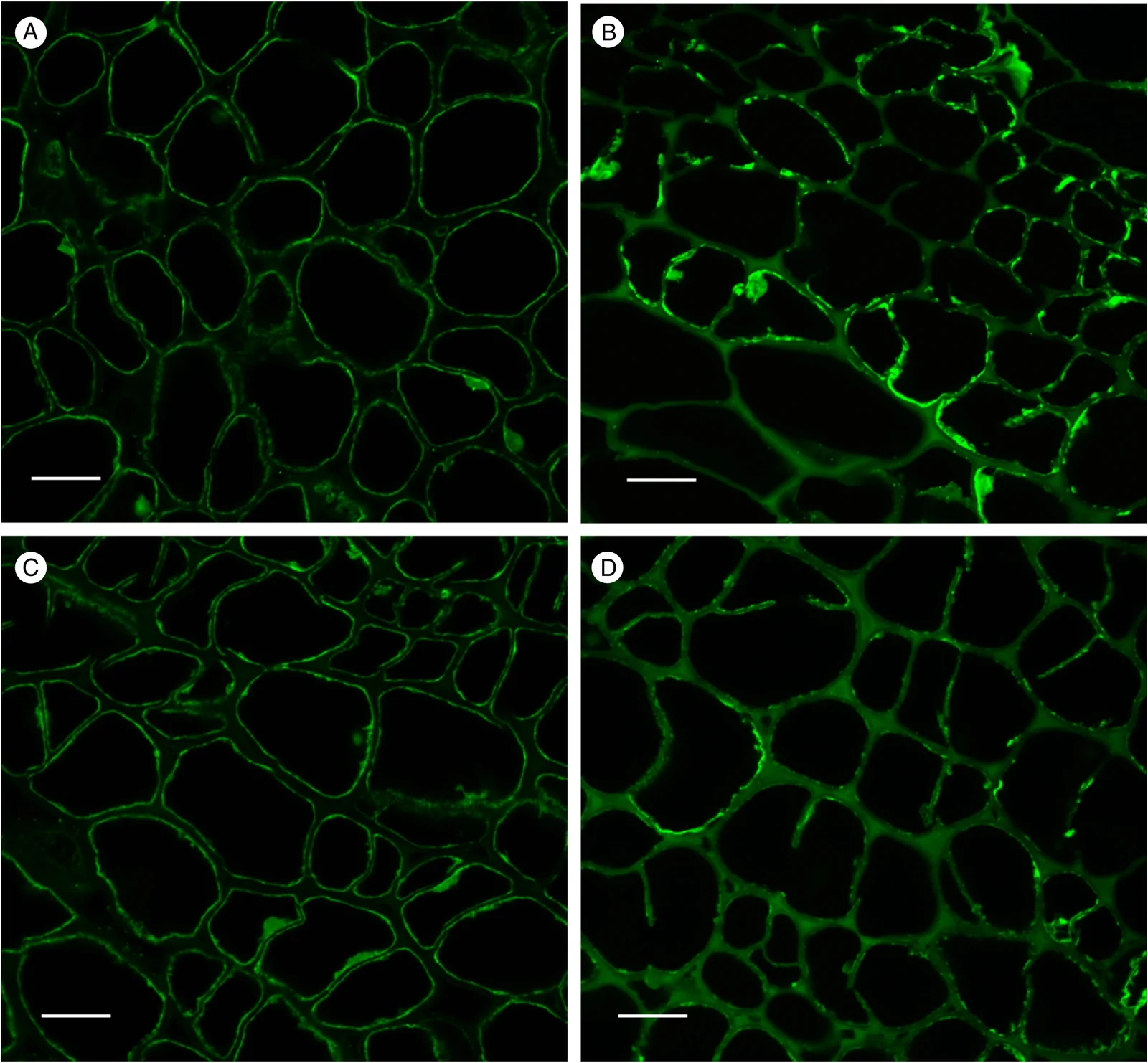
Root tissue from 6-week-old sugar beet plants of the variety SR98/2 immunolabeled with the rat IgM monoclonal antibodies LM19 and LM20, 4 d after inoculation: (A) LM19 healthy, (B) LM19 infected, (C) LM20 healthy and (D) LM20 infected. A goat anti-rat IgG secondary antibody conjugated to Alexa Fluor 488 dye was used as the fluorescent label. Imaged at 40× with a Nikon A1Rsi laser scanning confocal microscope. Scale bar = 25 µm.

## DISCUSSION

### Colonization and infection cushion formation

Hyphal density was greater on the susceptible sugar beet variety than on the resistant variety, a pattern previously hypothesized to contribute to increased infection sites and more severe disease symptoms (Bassi *et al*., 1979; Pannecoucque and Höfte, 2009; Zhang *et al*., 2016). In the present study, this increased hyphal density appeared to arise primarily from increased branching proliferation and hyphal aggregation. Similar associations between hyphal density and host susceptibility have been reported in tomato fruit (Bassi *et al*., 1979) and mungbean (Bashyal *et al*., 2018).

The observation that hyphae spread across the root surface without aligning to the anticlinal walls contrasts with infection patterns commonly described for *R. solani* in other hosts (Armentrout and Downer, 1987; Yang *et al*., 1992) yet is consistent with Ruppel’s (1973) original histopathology of RRCR and more recent observations in potato (Zhang *et al*., 2016). Because both potato tubers and sugar beet roots possess a periderm rather than an epidermis, this shared structural similarity may account for the absence of directed growth. Unlike epidermal cells, cork cells lack consistent linear orientation and uniformity, which may reduce directional cues for hyphal alignment (Albersheim *et al*., 2011).

Shortening of branch lengths leading to the formation of infection cushions has been reported in several pathosystems (Armentrout and Downer, 1987; Hofman and Jongebloed, 1988; Yang *et al*., 1992). However, in contrast to these reports, the shortened hyphae observed in the present study did not exhibit characteristic T-shaped branching. Notably, T-shaped branching was also absent during AG 3 infection of potato, and, as with the lack of directed growth, this pattern may reflect the differences between a periderm and an epidermis (Demirci and Döken, 1998; Zhang *et al*., 2016). Whether *R. solani* AG 2-2 forms T-shaped branches on roots with an epidermal surface, such as dry bean or soybean roots, warrants further investigation.

Root exudates have been implicated in hyphal recruitment, determination of invasion sites and infection cushion formation (Marshall and Rush, 1980; Badri and Vivanco, 2009; Kou and Naqvi, 2016; Lombardi *et al*., 2018). On the root surface, the frequent coincidence of infection cushion formation and hyphal overlap in the present study may reflect localized regions of exudate leakage. We hypothesize that exudate-rich sites may attract multiple hyphae independently to the same location, where infection cushions may subsequently form. Additional support for exudate-mediated recruitment comes from observations of hyphae accumulating around cracks or natural openings in the periderm, which are expected to be enriched in exudates (Fig. 2E).

Root system architecture is a plastic trait influenced by biotic and abiotic factors that can modulate branching and nutrient acquisition (Lynch and Brown, 2001; Badri and Vivanco, 2009). Root hairs and the region immediately behind the root cap are recognized as major sites of exudation, and both are concentrated within the root groove (Van Egeraat, 1975; Nguyen, 2003). Exudates have been shown to mediate interactions between plants and microbial communities of the rhizosphere (Doornbos *et al*., 2012), and elevated exudate production could promote colonization and guide hyphae to this vulnerable region. The potential role of exudates in *R. solani* AG 2-2 hyphal recruitment, infection structure development and host susceptibility warrants further study.

### Periderm penetration and early tissue invasion

Tissue colonization was evident within 3 d post-inoculation, and penetration of the periderm probably occurs within 24 h of hyphal contact with the root surface. Ruppel (1973) observed hyphae within the sugar beet cork cells by 2 d post-inoculation, although hyphal presence in the outer cortex and associated necrosis was not detected until 4 d post-inoculation. In potato infected by *R. solani* AG 3, periderm penetration was reported to occur in as little as 8 h post-inoculation, with most of these infections occurring through epidermal cracks and lenticels (Zhang *et al*., 2016). These findings imply that the periderm offers limited resistance to *R. solani* invasion. Similar to the colonization of cork cells by *Aspergillus nidulans* reported by Martins *et al*. (2014), the cork layer in sugar beet remained largely intact, with no evidence of extensive enzymatic breakdown of the periderm at this stage.

### Cortex invasion and the secondary cambium

Hyphae spread through the cortex primarily intracellularly but were also observed within parenchyma cells (Fig. 5). At sites where hyphae had breached the periderm and in older portions of the lesions, intercellular hyphae became more common and were often associated with fragmented cell walls (Fig. 4). The absence of infection cushions at most penetration sites, together with repeated observations of direct penetration (Fig. 3), indicates that infection cushions are not required for invasion, although we could not exclude the possibility that some cushions were present but not preserved during sectioning.

Because hyphae in early infection stages and in younger lesions progressed intracellularly, it could be hypothesized that invasion begins with penetration through the anticlinal walls. However, no conclusive evidence for this was observed, and invasion appeared to occur through multiple routes in an opportunistic manner. It is possible that hyphae penetrated cork cells at favourable sites and transitioned to intracellular growth after breaching the periderm.

In the smooth root region, the secondary cambium appeared to act as a temporary barrier to fungal progression. The basis for this constraint is unclear, as no obvious structural response was observed in the cambial tissues themselves. It is possible that the secondary cambium is less a true barrier and simply the less favourable route. Vascular cell walls are more complex than those of parenchyma and can become tough and dense without major changes in composition (Müller *et al*., 2003). The density and organization of vascular tissues may make access to the storage parenchyma more favourable.

In contrast, tissues within the root groove generally lacked this secondary cambium (Fig. 8). Substantial damage to lateral roots and surrounding tissues was observed within the root groove, where hyphae advanced more uniformly rather than laterally, as occurred when the secondary cambium was present. Coupled with the potential for elevated exudation, the root groove represents a vulnerable region for invasion.

### Cell wall response and callose deposition

Because of propidium iodide’s affinity for demethylated pectin (Rounds *et al*., 2011), we initially hypothesized that the increased fluorescence within the area of the lesion was due to the activity of pectin de-methylesterases, which could increase susceptibility of the cell wall to enzymatic degradation (Karr and Albersheim, 1970; Daher and Braybrook, 2015). However, immunofluorescence analyses did not support changes in pectin methylation state as the cause of increased propidium iodide binding. Instead, the enhanced signal appeared to reflect elevated autofluorescence associated with unknown localized changes in the cell wall (Fig. 9).

Aniline blue staining provided evidence that the increased autofluorescence in the lesions was associated with callose deposition (Li *et al*., 2023). Callose, a β-1,3-linked glucose polysaccharide, plays a role in cell wall formation and defence responses (Luna *et al*., 2011; Wang *et al*., 2021; Li *et al*., 2023). Deposition of calloses between the plasma membrane and the primary cell wall can reinforce the cellulose matrix and contribute to cell wall thickening (Wang *et al*., 2021). Additionally, callose accumulation at plasmodesmata can regulate cell wall permeability and may restrict the intracellular movement of effector molecules, thereby mitigating pathogen impact on plant physiology (Wang *et al*., 2022; Li *et al*., 2023).

Observations from the present study support both structural reinforcement and restricted intracellular movement as potential mechanisms of resistance in the sugar beet variety SR98/2. Cell walls at the lesion margin were visibly thicker than those in healthy tissue (Fig. 9) and staining with aniline blue supported the hypothesis that callose deposition was also involved. Fluorescence signal differed markedly between the healthy and symptomatic side of the callose band, consistent with callose deposits impeding the diffusion of pathogen-derived molecules. This dual role, physical reinforcement and molecular containment, may contribute to reduced disease severity in SR98/2. Ruppel (1973) reported that no mechanical barrier developed in advance of invading hyphae in the resistant cultivar examined in that study, although callose was not specifically assessed. However, cell wall thickening was observed by Ruppel (1973), consistent with the present study and this comparison raises the possibility of a shared structural response among resistant cultivars. Further work is needed to determine the prevalence and regulation of callose deposition or other cell wall factors in sugar beet resistance.

### Pectin modifications and enzymatic activity

*Rhizoctonia solani* AG 2-2 appears to employ multiple strategies for host tissue penetration, adapting to diverse environmental and anatomical contexts through a barrage-style strategy that can overcome barriers to invasion. A previous genomic analysis of *R. solani* AG 2-2 revealed a large repertoire of secreted proteins, including 1097 putative carbohydrate-active enzymes (CAZymes), many of which are predicted to function in cell wall degradation (Wibberg *et al*., 2016). This enzymatic diversity supports the hypothesis that the fungus can initiate infection through various routes, reflecting substantial genomic plasticity in pathogenicity and virulence factors (King *et al*., 2011; Wibberg *et al*., 2016). Such enzymatic flexibility might affect the ability of fungal pathogens to adapt to different hosts and support multiple infection strategies that exploit a wide range of plant defence vulnerabilities (King *et al*., 2011).

The loss of LM19 and LM20 antibody binding sites from the cell wall indicates degradation of both methylated and demethylated pectin epitopes (Figs 12 and 13). Although this degradation occurred in both the resistant and susceptible varieties, it appeared to progress more rapidly in the susceptible variety, C869, though no quantitative analysis was performed. In C869, unesterified pectin was largely eliminated from affected cell walls within 4 d of inoculation, whereas in the resistant variety, unesterified pectin accumulated along the inner surface of the cell wall (Fig. 13). This accumulation may reflect the localized buildup of oligogalacturonides (OGs), which are recognized as damage-associated molecular patterns (DAMPs; Cervone *et al*., 1989; Ridley *et al*., 2001; Pontiggia *et al*., 2015). Oligogalacturonides are known to activate multiple defence responses, including cell wall reinforcement (Bellincampi *et al*., 2014), callose deposition (Flors *et al*., 2008), lignification (Xu *et al*., 2011; Eynck *et al*., 2012) and the formation of cross-links between cell wall components (Passardi *et al*., 2004; Deepak *et al*., 2010). The evidence for the accumulation of pectin aggregates in the resistant variety SR98/2 is consistent with activation of the callose reinforcement pathway and may reflect a broader defence response.

The accumulation of OGs can be influenced by interactions between fungal polygalacturonases (PGs) and host-derived polygalacturonase-inhibiting proteins (PGIPs; De Lorenzo *et al*., 1994; Cook *et al*., 1999; Davidsson *et al*., 2017). Because polygalacturonase enzymes exhibit specificity relative to structural context (D’Ovidio *et al*., 2004), the secretion of multiple isoforms by fungal pathogens may reduce OG fragments to sizes that fail to elicit a defence response (Scala *et al*., 1980; Benedetti *et al*., 2015). Inhibition of specific isoforms by PG–PGIP interactions may promote the accumulation of OGs of sufficient size to trigger defence responses, thereby enhancing cell wall reinforcement and signalling cascades that contribute to host resistance.

Several PGIPs have been identified in sugar beet (Li and Smigocki, 2016) and in other crops where they contribute to resistance against fungal pathogens (Kalunke *et al*., 2015; Liu *et al*., 2016; Li and Smigocki, 2018; Chen *et al*., 2019). These proteins may be induced by wounding (Li and Smigocki, 2016) or accumulation of OGs (D’Ovidio *et al*., 2004) and play a role in plant innate immunity by modulating specific types of enzymatic activity (Di Matteo *et al*., 2006; Federici *et al*., 2006). Investigating PGIP gene expression in SR98/2 and other resistant sugar beet lines could provide insight into resistance mechanisms and identify targets for breeding programmes aimed at enhancing disease resistance.

In addition to polygalacturonase activity, the degradation of highly esterified pectin, evidenced in the present study by reduced binding of the LM20 antibody, implicates the activity of pectin lyase, currently the only known enzyme capable of degrading highly methylated pectin (Yadav *et al*., 2009). Pectin lyase has been isolated from *R. solani* AG 2-2 and shown to exhibit greater activity on sugar beet tissue than polygalacturonase, both in culture and in infected sugar beet samples (Bugbee, 1990). Further support for a role of pectin lyase in pathogenesis comes from the *Rhizoctonia*-resistant cultivar FC712 (Panella, 2001), in which Bugbee (1993) reported reduced pectin lyase activity compared to susceptible varieties. This reduction in activity was attributed to a host-derived proteinaceous pectin lyase inhibitor that may offer an additional genetic trait that can contribute to resistance against pectin degradation.

## CONCLUSIONS

*Rhizoctonia solani* AG 2-2 utilizes multiple routes to penetrate sugar beet roots, reflecting a flexible infection strategy shaped by both anatomical features and environmental context. Although early infection behaviour differs from that reported for above-ground tissues and on other hosts (Bassi *et al*., 1979; Marshall and Rush, 1980; Hofman and Jongebloed, 1988; Pannecoucque and Höfte, 2009; Bashyal *et al*., 2018), its similarity to the behaviour of AG 3 on potato tubers probably reflects common infection dynamics associated with periderm-based tissues (Zhang *et al*., 2016). While the periderm offered little resistance to invasion, the outer cambium temporarily restricted hyphal advance, leading to lateral spread and producing the shallow, ladder-like lesions often observed for RRCR. In contrast, tissues in the root groove lack this barrier and were more rapidly colonized. Enhanced callose deposition in the resistant cultivar SR98/2 was associated with limited colonization.

Necrosis consistently preceded hyphal advance, as previously reported (Ruppel, 1973), suggestive of the involvement of secreted cell-wall-degrading enzymes (CWDEs). The extensive CAZyme repertoire reported for *R. solani* AG 2-2 (Wibberg *et al*., 2016) aligns with our observations of cellulose, pectin and methylated pectin degradation (Scala *et al*., 1980; Bugbee, 1990; El-Samawaty *et al*., 2008). Comparative studies of CWDEs across AG 2-2 isolates and other anastomosis groups could clarify whether enzymatic diversity helps shape host specificity, virulence and resistance strategies.

The sugar beet variety SR98/2 exhibits strong resistance to RRCR at both the seedling and adult stage and was therefore an appropriate choice for inclusion in the present study. As strong resistance has been reported to be consistent in multiple geographical locations and diverse *R. solani* AG 2-2 strains (e.g. Ruppel *et al*., 1979; Buddemeyer and Marlander, 2005; Strausbaugh *et al*., 2013), the results of the current study are likely to be relevant for a range of materials. However, some variability in response by strain has been reported in varieties with weaker resistance (Strausbaugh *et al*., 2013) and these may rely on different or a subset of resistance mechanisms, making them candidates for future comparative studies.

## AI ASSISTANCE

The use of AI in the preparation of this paper was limited to basic editorial functions such as correcting grammatical and spelling errors, and for reference formatting.
